# Supplementary material for: Genomic Consequences of Fragmentation in the Endangered Fennoscandian Arctic Fox (Vulpes lagopus)
Source: Genes (Basel). 2022 Nov 15;13(11):2124. doi: 10.3390/genes13112124 (PMC9690288; doi:10.3390/genes13112124)
Supplement: Supplementary file 1 [file genes-13-02124-s001.zip › genes-1931147-supplementary.pdf]

Supplementary information

Genomic Consequences of Fragmentation in the Endangered Fennoscandian Arctic Fox (*Vulpes lagopus*)

Christopher A. Cockerill, Malin Hasselgren, Nicolas Dussex, Love Dalén, Johanna von Seth, Anders Angerbjörn, Johan F. Wallén, Arild Landa, Nina E. Eide, Øystein Flagstad, Dorothee Ehrich, Aleksandr Sokolov, Natalya Sokolova and Karin Norén

Table S1: Genome-based individual genetic variation in the sample areas of northern Fennoscandia and Siberia. Sequencing coverage; Het/kb = heterozygous sites per 1000 bp; F_{ROH} > 100kb = proportion of genome contained within ROH larger than 100 kb; F_{ROH} >2 Mb = proportion of genome contained in ROH larger than 2 Mb; FROH >8Mb = proportion of genome contained in ROH larger than 8 Mb.

**Sample**	**Sample area**	**Coverage**	**Het/kb**	**F_{ROH} >100 kb**	**F_{ROH} >2 Mb**	**F_{ROH} >8 Mb**	**ENA Project ID**
0413	Vindelfjällen		1.75	0.200	0.120	0.077	PRJEB55788
0416	Vindelfjällen		1.71	0.238	0.154	0.107	PRJEB55788
0592	Vindelfjällen		1.86	0.168	0.082	0.038	PRJEB55788
0596	Vindelfjällen		1.82	0.169	0.086	0.029	PRJEB55788
0760	Vindelfjällen		1.45	0.354	0.286	0.249	PRJEB55788
0883	Vindelfjällen		1.71	0.223	0.122	0.059	PRJEB55788
1491	Vindelfjällen		1.87	0.184	0.103	0.047	PRJEB55788
1607	Vindelfjällen		1.78	0.213	0.141	0.091	PRJEB55788
1714	Vindelfjällen		1.80	0.208	0.116	0.065	PRJEB55788
1967	Vindelfjällen		1.82	0.177	0.092	0.027	PRJEB55788
8917	Vindelfjällen		1.80	0.202	0.130	0.053	PRJEB55788
11113	Vindelfjällen		1.63	0.271	0.205	0.151	PRJEB55788
11119	Vindelfjällen		1.65	0.263	0.182	0.117	PRJEB55788
14170	Vindelfjällen		1.81	0.194	0.122	0.076	PRJEB55788
15158	Vindelfjällen		1.75	0.224	0.130	0.079	PRJEB55788
0875	Arjeplog		1.43	0.382	0.308	0.232	PRJEB55788
0877	Arjeplog		1.41	0.365	0.295	0.228	PRJEB55788
1976	Arjeplog		1.71	0.239	0.154	0.101	PRJEB55788
14176	Arjeplog		1.66	0.272	0.189	0.144	PRJEB55788
14182	Arjeplog		1.61	0.304	0.219	0.156	PRJEB55788
18110	Arjeplog		1.71	0.241	0.153	0.091	PRJEB55788
19088	Arjeplog		1.77	0.227	0.142	0.091	PRJEB55788
19089	Arjeplog		1.87	0.238	0.160	0.103	PRJEB55788
19091	Arjeplog		1.84	0.162	0.082	0.022	PRJEB55788
19099	Arjeplog		1.82	0.192	0.112	0.067	PRJEB55788
19104	Arjeplog		1.80	0.203	0.122	0.063	PRJEB55788
5973	Dividalen		1.80	0.203	0.110	0.065	PRJEB55788
5965	Reisa Nord		1.84	0.167	0.079	0.025	PRJEB55788
5966	Saltfjellet		1.72	0.143	0.056	0.017	PRJEB55788
2692	Varanger		1.86	0.179	0.108	0.064	PRJEB55788
2230	Taymyr		2.07	0.034	0.009	0.004	PRJEB55788
2232	Taymyr		2.07	0.035	0.009	0.004	PRJEB55788
5003	Taymyr		2.07	0.027	0.004	0	PRJEB55788
5011	Taymyr		2.07	0.022	0	0	PRJEB55788

6220	Taymyr	2.09	0.023	0.004	0	PRJEB55788
3108	Yamal	2.06	0.048	0.024	0.016	PRJEB55788
3112	Yamal	2.1	0.022	0.002	0	PRJEB55788
3120	Yamal	2.07	0.038	0.009	0	PRJEB55788
5974	Yamal	1.87	0.040	0.012	0	PRJEB55788
5967	Kola	1.81	0.010	0.051	0.029	PRJEB55788
9472	Indigirka	2.05	0.037	0.010	0	PRJEB55788
9474	Faddeyevsky Island	2.09	0.033	0.007	0.006	PRJEB55788
8004	Wrangel Island	2.1	0.032	0.007	0	PRJEB55788

Table S2: Results of the post hoc Dunn's tests showing significance of difference of genome-wide heterozygosity among northern Sweden, northern Norway, Kola, Yamal, Taymyr and eastern Siberia. Significant values in bold with * P<0.05, ** P<0.01, ***P<0.001.

FROH	Eastern Siberia	Kola	Northern Norway	Northern Sweden	Taymyr
Kola	NS	-	-	-	-
Northern Norway	NS	NS	-	-	-
Northern Sweden	**0.0253***	NS	NS	-	-
Taymyr	NS	NS	NS	**0.00311****	-
Yamal	NS	NS	NS	**0.0253***	NS

Table S3: Results of the post hoc Dunn's tests, showing significance of difference in inbreeding due to common ancestors less than 10 generations ago among northern Sweden, northern Norway, Kola, Yamal, Taymyr and eastern Siberia. Significant values in bold with * P<0.05, ** P<0.01, ***P<0.001.

FROH	Eastern Siberia	Kola	Northern Norway	Northern Sweden	Taymyr
Kola	NS	-	-	-	-
Northern Norway	NS	NS	-	-	-
Northern Sweden	**0.0321***	NS	NS	-	-
Taymyr	NS	NS	NS	**0.00211****	-
Yamal	NS	NS	NS	**0.00702****	NS

Table S4: Results of the post hoc Dunn's tests, showing significance of difference in inbreeding due to common ancestors up to 45 generations back among northern Sweden, northern Norway, Kola, Yamal, Taymyr and eastern Siberia. Significant values in bold with * P<0.05, ** P<0.01, ***P<0.001.

FROH	Eastern Siberia	Kola	Northern Norway	Northern Sweden	Taymyr
Kola	NS	-	-	-	-
Northern Norway	NS	NS	-	-	-
Northern Sweden	**0.033***	NS	NS	-	-
Taymyr	NS	NS	NS	**0.000475*****	-
Yamal	NS	NS	NS	**0.0189***	NS

Table S5: Results of the post hoc Dunn's tests showing significance of difference in inbreeding due to common ancestors 850 generations among northern Sweden, northern Norway, Kola, Yamal, Taymyr and eastern Siberia. Significant values in bold with * P<0.05, ** P<0.01, ***P<0.001.

F_{ROH}	Eastern Siberia	Kola	Northern Norway	Northern Sweden	Taymyr
Kola	NS	-	-	-	-
Northern Norway	NS	NS	-	-	-
Northern Sweden	**0.0305***	NS	NS	-	-
Taymyr	NS	NS	NS	**0.00053*****	-
Yamal	NS	NS	NS	**0.0232***	NS

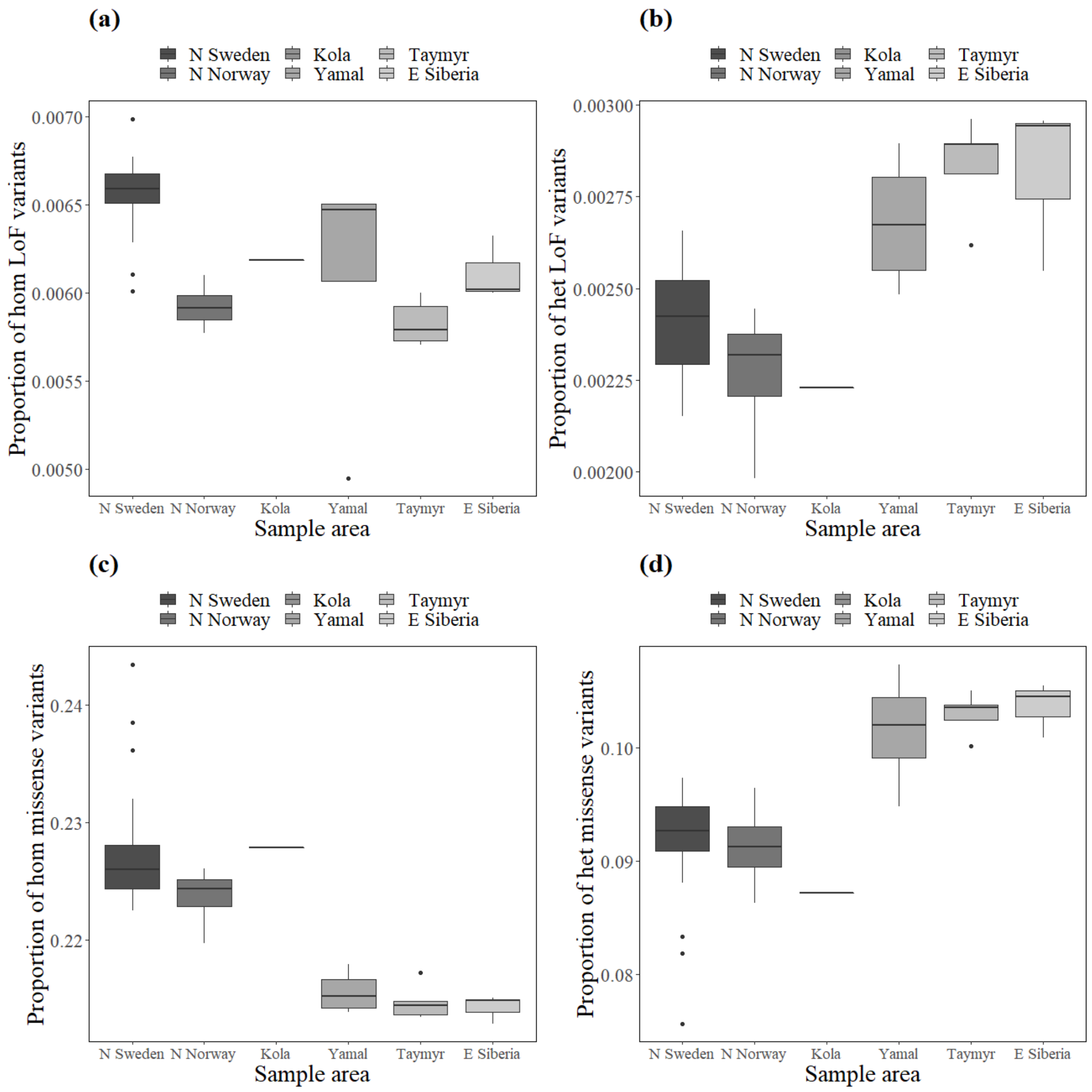

Figure S1: Proportion of deleterious variants in arctic foxes of the northern Sweden, northern Norway, Kola, Yamal, Taymyr and eastern Siberia sample areas; separated into (a) homozygous loss of function variants (LoF), (b) heterozygous LoF variants, (c) homozygous missense variants, and (d) heterozygous missense variants. Proportion calculated as the number of each variant type divided by total variants. The horizontal bar shows the median, the boxes show the 25–75% interquartile range and the whiskers show the whole range.
